# Cancer-Associated Fibroblasts: Clinical Applications in Imaging and Therapy

**DOI:** 10.3390/tomography11120143

**Published:** 2025-12-17

**Authors:** Neda Nilforoushan, Ashkan Khavaran, Maierdan Palihati, Yashvi Patel, Anna O. Giarratana, Jeeban Paul Das, Kathleen M. Capaccione

**Affiliations:** 1 Department of Radiology, Columbia University Irving Medical Center, New York, NY 10032, USA; nn2700@cumc.columbia.edu (N.N.); mp4450@cumc.columbia.edu (M.P.); pately8@tcnj.edu (Y.P.); 2 Faculty of Medicine, University of Freiburg, 79110 Freiburg, Baden Wurttemberg, Germany; ashkan.khavaran@fccc.edu; 3 Department of Pathology, Fox Chase Cancer Center, Temple University, Philadelphia, PA 19111, USA; 4 Department of Radiology, University of Wisconsin-Madison, Madison, WI 53792, USA; agiarratana@uwhealth.org; 5 Department of Radiology, Memorial Sloan Kettering Cancer Center, New York, NY 10065, USA; dasj@mskcc.org

**Keywords:** cancer associated fibroblasts (CAFs), tumor microenvironment (TME), fibroblast activation protein (FAP), heterogeneity, stromal targeting, imaging biomarkers, radioligand therapy

## Abstract

Cancer-associated fibroblasts (CAFs) are an abundant and diverse cell population within tumor microenvironments of solid tumors. Multiple subtypes of CAFs, defined by molecular and functional markers, have been described in the literature. CAFs contribute to tumor progression by remodeling the extracellular matrix, promoting immune evasion, and supporting angiogenesis and metastasis. Fibroblast activation protein (FAP) is a transmembrane serine protease minimally expressed in normal adult tissues but significantly upregulated in certain subtypes of CAFs across many solid tumors. High levels of FAP have been associated with poor prognosis in various cancers. FAP has increasingly emerged as a promising target for both imaging and therapy. Multiple FAP-targeting strategies, such as small molecules, monoclonal antibodies, drug conjugates, and radiolabeled ligands, are currently being investigated in preclinical and early clinical settings. This review provides a clinically focused overview of CAFs in the tumor microenvironment, highlighting key fibroblast markers, their associations with prognosis across various tumor types, and their utility in radiologic imaging and targeted therapy. We also discuss the potential of non-FAP fibroblast targeting molecules and the clinical rationale for more selective, subtype-specific strategies. By examining fibroblast biology through a radiologist’s lens, we aim to explore the evolving role of stromal targeting in imaging and the treatment of solid tumors.

## Simple Summary

Cancer-associated fibroblasts are supportive cells found within the environment of solid tumors. These cells help tumors to grow, spread, and resist treatments by actively changing the tumor environment and modulating immune response. In recent years, scientists have discovered that fibroblasts vary in type and function, and some can be specifically targeted for diagnosis and therapy. This review explores the many roles of these cells in cancer and how they can be used for imaging and treatment, and highlights promising new strategies that may improve cancer detection and open the door to more effective treatments.

## 1. Introduction

Cancer-associated fibroblasts (CAFs) are stroma cells abundantly present in the tumor microenvironment (TME) [1]. CAFs can acquire tumor-promoting phenotypes which significantly impact cancer pathology [2,3,4]. Rather than serving as a passive scaffold, CAFs actively reshape the structural, biochemical, and immunological landscape of tumors, thus facilitating cancer progression, therapy resistance, and immune evasion [5,6]. CAFs constitute a substantial portion of the stromal compartment in most solid tumors, and their abundance is generally linked to poor clinical prognosis, advanced disease stages, and therapeutic resistance [7]. CAFs uniquely bridge mechanical remodeling and paracrine signaling through their secretion of extracellular matrix (ECM) proteins, cytokines, and growth factors [8,9], modulating both cellular behavior and physical properties of the tumor.

Recent advances in single-cell and spatial transcriptomics have revealed previously unrecognized cell types within the TME, including the discovery that CAFs are not a uniform population but instead comprise functionally and transcriptionally distinct subtypes with context-dependent roles [10]. This heterogeneity, along with their prevalence in tumors, highlights the importance of CAFs as both biomarkers and therapeutic targets [11,12,13]. Recently, attempts to modulate CAFs have included inhibiting key pathways involving TGF-β or targeting specific markers like Fibroblast Activation Protein (FAP) [3]. The future of CAF-directed therapy likely resides in precision medicine that selectively inhibits specific pro-tumorigenic CAF subsets [2].

Given the rapid expansion of the CAF literature and the clinical momentum surrounding FAP-targeted agents, a consolidated and up-to-date synthesis is needed. To our knowledge, no recent review has integrated the latest clinical developments in FAP imaging and theranostics, with an updated overview of CAF heterogeneity, molecular function, and emerging non-FAP stromal targets. In this review, we summarize current knowledge on CAF biology, outline their mechanistic contribution to tumor progression, and provide a clinically oriented survey of CAF-directed therapeutic strategies, including, but not limited to, FAP-targeted imaging, radioligand therapy, and stromal modulation. By bridging fundamental stromal biology with evolving clinical applications, this work aims to offer a comprehensive and timely reference for researchers and clinicians in this field.

## 2. Cancer-Associated Fibroblasts and the Tumor Microenvironment

Once activated, CAFs play a central role in shaping the TME through reciprocal interaction with cancer cells and surrounding stromal elements [14]. By secreting cytokines, chemokines, and ECM components, CAFs contribute to a protumorigenic niche that promotes malignancy, immune evasion, and resistance to therapy [8,15]. The following sections outline the diverse, context-dependent, roles of CAFs in modulating tumor behavior, immune responses, metabolism, and treatment outcome.

*Physical Tumor Microenvironment*: CAFs are central in remodeling the ECM by producing collagens, fibronectin, and hyaluronic acid, leading to desmoplasia which is a stiff, fibrotic stroma that elevates interstitial pressure, enhances mechanotransduction, and facilitates tumor invasion and epithelial-to-mesenchymal transition (EMT) through integrin and focal adhesion signaling [16,17].

*Malignancy Induction and Tumor Progression*: Disfunction of the resident tissue fibroblasts can in and of itself lead to malignancy [18]. Pathways implicated in fibroblast induced malignancy include the loss of TGF-β pathway regulation in fibroblasts, activation of the JAK-STAT3 pathway in gastrointestinal tumors and blockage of the CXCR4 receptor in mamillary tissue [18,19,20,21]. Once transformed into CAFs, these cells promote tumor progression through paracrine signaling and ECM remodeling. They secrete growth factors such as TGF-β, HGF, EGF, and IL-6, which drive malignant transformation, therapy resistance, and EMT [22]. CAFs also support angiogenesis via VEGF, CXCL12, and PDGF, facilitating new blood vessel formation and metastatic spread [23,24]. In addition, CAF-derived proteins (e.g., ephrins, cadherins, tenascin-C, and thrombospondin-1) and exosomes contribute to cell–cell interactions and promote both local invasion and distant colonization [25,26,27,28].

*Immune Modulation*: CAFs suppress anti-tumor immunity by secreting immunosuppressive cytokines (TGF-β, IL-10), expressing checkpoint ligands (PD-L1, PD-L2, FASL), and altering chemokine gradients (CXCL12) to exclude cytotoxic T cells [29,30,31,32]. They promote expansion of regulatory T cells (Tregs) and M2-polarized macrophages via IL-6, M-CSF, CCL2, and IL-8, and impair dendritic cell maturation through IL-10 and TGF-β. CAFs also secrete amyloid-β, recruiting tumor-promoting neutrophils [33,34,35,36,37,38].

*Metabolic Effects*: CAFs support tumor energetics through bidirectional metabolic interactions with malignant cells. CAFs absorb lactate produced by tumor glycolysis and modulate the pH and nutrient composition of the TME [39,40]. They secrete additional metabolites such as alanine to sustain tumor growth under nutrient stress [41].

*Therapy Resistance*: CAFs contribute to resistance against chemotherapy and targeted therapies through multiple mechanisms. They transfer exosomal RNAs and secrete cytokines like IL-6 that enhance tumor plasticity, leading to resistance to agents such as oxaliplatin and cisplatin [42,43]. Additionally, CAF-induced desmoplasia increases tissue stiffness and reduces vascular perfusion, hindering drug delivery [44].

**Figure 1:**
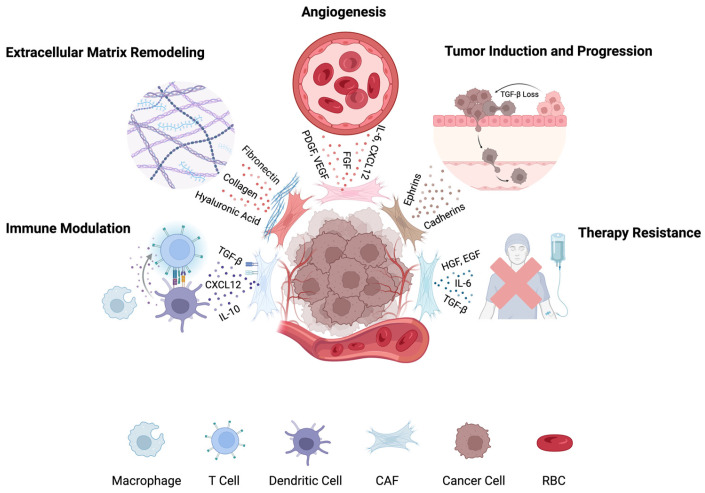
Roles of cancer-associated fibroblasts (CAFs) in the tumor microenvironment. CAFs contribute to tumor progression by remodeling the ECM, suppressing immune responses, promoting angiogenesis, enhancing metastasis and proliferation, and driving therapy resistance. Distinct CAF subtypes mediate these processes through secretion of cytokines, growth factors, and matrix proteins. Figure created in BioRender.

A schematic overview of CAFs role in the TME is illustrated in Figure 1.

## 3. Heterogeneity of CAFs

Importantly, CAFs do not represent a uniform population. Recent advances in single-cell RNA sequencing (scRNA-seq), spatial transcriptomics, and lineage tracing have revealed that CAFs consist of multiple phenotypically and functionally distinct subtypes [8,16]. These include myofibroblastic CAFs (myCAFs), which are contractile and matrix-depositing; inflammatory CAFs (iCAFs), which secrete cytokines and chemokines [17,45]; antigen-presenting CAFs (apCAFs), which express MHC class II molecules [12]; vascular CAFs (vCAFs) which are located in perivascular regions; and matrix-remodeling CAFs (mCAFs) which are enriched for ECM-associated genes [46,47]. Each of these subtypes plays different roles in tumor progression, immune modulation, and therapy resistance [7].

CAF subtype composition and spatial distribution are highly context-dependent and may vary with tumor type, stage, anatomical site, and therapy exposure. Some subsets may demonstrate protumorigenic effects, while others may restrain tumor growth or modulate immune responses in more complex ways [8,48]. This functional heterogeneity underscores the need for subtype-specific therapeutic strategies, as global CAF depletion has yielded paradoxical effects in preclinical models [49,50]. Distinct protein expression pattern within the tumor microenvironment have also been shown to influence tumor progression, immune landscape, and therapeutic responsiveness, highlighting the importance of molecular heterogeneity in guiding targeted interventions [51,52].

### CAF Markers and Expression Patterns

Identifying CAFs remains difficult due to the lack of a single definitive marker identifying the cell type and their heterogenous and plastic nature. They are typically defined by a combination of positive markers reflecting activation or differentiation, and negative markers to exclude epithelial (EpCAM), endothelial (CD31), and hematopoietic (CD45) lineages [53,54,55].

Among positive markers, α-smooth muscle actin (α-SMA) is a hallmark of myCAFs, which are contractile and contribute to matrix deposition and desmoplasia. However, α-SMA is also expressed by pericytes and vascular smooth muscle cells, limiting specificity [56,57]. Fibroblast activation protein (FAP), a membrane-bound serine protease enriched in tumor-associated stroma, is widely used due to its minimal expression in normal adult tissues and its role in matrix remodeling and immune modulation. Still, FAP expression varies by CAF subtype and tumor stage [58,59,60,61]. Fibroblast-specific protein 1 (FSP1/S100A4) marks a broader CAF population associated with motility and metastasis, including non-proliferating or FAP-negative subsets; however, it is also expressed in certain immune and endothelial cells [62,63,64]. Other broadly used markers such as platelet-derived growth factor receptors (PDGFR-α/β) and vimentin (VIM) reflect mesenchymal identity but exhibit cross-lineage expression, being present in pericytes, endothelial cells, and EMT-activated epithelial cells [53,54,65,66].

Recent research has identified emerging markers that further delineate functional CAF subsets. Leucine-rich repeat-containing protein 15 (LRRC15) marks TGF-β–responsive CAFs enriched in matrix remodeling and immune suppression, particularly in lung, breast, and pancreatic tumors [67,68]. G-protein coupled receptor 77 (GPR77) identifies CAFs that promote cancer stemness and chemoresistance, correlating with poor prognosis [69]. ApCAFs express MHC class II molecules such as HLA-DRA, and CD74 and may play a role in local immune tolerance within the tumor [12,70]. Additional context-dependent markers include CD90 (Thy-1), podoplanin (PDPN), and tenascin-C (TNC), which are associated with immune modulation, stromal remodeling, and therapy response in specific tumor types [71,72,73,74].

No single marker sufficiently captures the complexity of CAF identity. A combinatorial marker panel approach, informed by tumor type, anatomical context, and functional state, along with integration of multi-omic approaches such as single-cell RNA sequencing remains essential to resolve CAF heterogeneity [56,72]. A comparative summary of key CAF markers, their associated subpopulations, biological roles, tumor-specific expression, and interpretive limitations is provided in Table 1.

## 4. Strategies to Attenuate and Reprogram CAF Activity

CAFs are increasingly recognized as promising imaging and therapy targets due to their role in tumor progression, immune evasion, and therapy resistance [79]. High CAF abundance is associated with poor prognosis and reduced treatment efficacy in many contexts [80,81]. While early strategies aimed at broadly depleting CAF populations, these untargeted approaches have not consistently improved outcomes, and in some cases, have led to accelerated tumor growth and invasion [49,50]. Recognizing CAF heterogeneity has shifted the focus toward selectively targeting pro-tumorigenic subtypes and modulating CAF signaling pathways at various stages of tumor development and immune interaction, rather than broad depletion [3].

To this end several approaches have been explored. One strategy seeks to block the initial activation of quiescent resident fibroblasts into CAFs by targeting upstream signaling pathways such as FGFR, Hedgehog, and TGF-β, though clinical benefit has been inconsistent [82,83,84,85,86]. For example, TGF-β is a central regulator of CAF differentiation, converting quiescent fibroblasts or bone marrow–derived mesenchymal stem cells into activated, tumor-promoting CAFs. In breast cancer models, this transition involves the myeloid zinc finger 1 (MZF1)/TGF-β axis and is associated with increased expression of CAF markers and increased tumor-promoting activity. Inhibition of TGF-β signaling reduces both CAF activation and tumor growth, highlighting its therapeutic promise despite challenges related to its broad physiological functions [76,87].

Other interventions disrupt CAF effector functions, for instance by using FAK or ROCK inhibitors to reduce matrix remodeling, or by targeting PDGFR, LRRC15, FSP-1, and immunomodulatory cytokines such as IL-6 and CXCLs [88,89,90,91,92,93]. Additional strategies include stromal depletion (e.g., PEGPH20 targeting hyaluronic acid) and losartan, an angiotensin II blocker that modulates the fibrotic TME, have shown preclinical promise but limited clinical impact [94,95,96].

Emerging research has revealed the importance of metabolic reprogramming in CAFs. These cells frequently undergo a glycolytic shift, termed the “reverse Warburg effect”, in which non-cancerous stromal cells such as CAFs metabolize glucose via aerobic glycolysis, producing lactate, even in the presence of oxygen. This phenomenon is metabolically similar to the classic “Warburg effect”, which occurs within cancer cells themselves, but in reverse Warburg effect, energy-rich metabolites, such as lactate and pyruvate, produced by CAFs are transferred to adjacent tumor cells to fuel mitochondrial oxidative phosphorylation [97]. Tumor-derived factors such as TGF-β and HIF-1α drive this metabolic reprogramming in CAFs. The lactate secreted not only supports cancer cell metabolism but also enhances immunosuppression by promoting IL-6 secretion that impairs CD8^+^ T-cell cytotoxicity [98,99,100]. Pharmacologic inhibition of lactate production using LDHA inhibitors, such as FX11, has been shown to restore antitumor immune responses and enhance the efficacy of PD-L1 blockade [101]. Beyond glycolysis, CAFs also exhibit enhanced glutamine and lipid metabolism that support tumor growth and immune evasion. Targeting these metabolic dependencies offers an opportunity to disrupt stromal–tumor crosstalk and synergize with existing immunotherapies [102].

Another therapeutic approach aims not to eliminate CAFs, but to revert them to a quiescent or tumor-suppressive phenotype [79]. Vitamin D and vitamin A analogs have shown promise in this context by reprogramming activated fibroblasts into a more differentiated, stellate-like state, particularly in pancreatic cancer models [103,104,105]. Similarly, inhibition of HSP70 has demonstrated efficacy in suppressing the pro-tumorigenic functions of CAFs, potentially through disruption of stress response pathways and attenuation of inflammatory signaling [106]. HSP90 has also emerged as a promising target, as it supports ECM remodeling, tumor growth and angiogenesis, which are key processes in sustaining CAF-mediated tumor progression [107,108].

Retinoic acid (RA) signaling is a key regulatory axis in fibroblast differentiation, but its activity can be attenuated by the nuclear receptor co-repressor RIP140. Chromatin immunoprecipitation studies have shown that RIP140 competes with coactivator complexes at RA-targeted promoters, delaying histone acetylation and recruitment of coactivators such as p300/CREB-binding protein-associated factor (P/CAF). The presence of RIP140 suppresses RA-induced gene expression despite ligand binding, and RA treatment induces dynamic assembly of retinoic acid receptor (RAR) coregulator complexes over time. These findings suggest that modulating RIP140 may enhance the transcriptional output of RA signaling in the tumor stroma [109].

While these strategies are supported by encouraging preclinical data, clinical trials evaluating CAF reprogramming remain in early stages. Results have so far been mixed, underscoring the need for precise therapeutic modulation and better characterization of CAF subpopulations to predict response.

## 5. FAP-Expressing CAFs: A Targetable Subset for Imaging and Therapy

FAP is a membrane-bound serine protease highly expressed in a subset of CAFs across many epithelial cancers, while showing minimal expression in normal tissues [110]. This selective expression supports its use as a tumor-restricted marker for both therapeutic and diagnostic purposes. FAP-positive CAFs contribute to tumor progression through ECM remodeling, immunosuppression, and angiogenesis, particularly in desmoplastic and therapy-resistant cancers [2,111,112]. Despite its variable expression among CAF subtypes and tumor stages, the high tumor-to-background contrast of FAP makes it a promising candidate for imaging and drug targeting [113]. In the following sections, we explore the correlation of FAP^+^ CAFs with prognosis, the current state of FAP-targeted therapeutic approaches, and emerging clinical applications in molecular imaging and drug delivery.

### 5.1. Prognostic Implications of FAP Expression Across Tumor Types

FAP expression on CAFs has been explored as a prognostic biomarker in several cancers, though its significance varies by tumor type. In general, high stromal FAP levels correlate with poorer outcomes, especially in aggressive or therapy-resistant tumors [114,115].

*Pancreatic and colorectal cancer*: Elevated FAP expression correlates with shorter overall and disease-free survival, higher recurrence, and increased metastatic potential [116,117].

*Breast cancer*: The prognostic significance of FAP is more variable. Some studies have identified a negative prognostic association, particularly in hormone receptor–negative subtypes, while others have reported no clear correlation or even improved survival in certain cohorts [118,119,120].

*Non-small cell lung cancer (NSCLC)*: Paradoxically, higher stromal FAP levels have been linked to improved prognosis, reinforcing the need for tumor-specific interpretation in clinical settings [121].

*Ovarian, hepatocellular carcinoma and neuroendocrine pancreatic tumors*: High FAP is associated with advanced stage, treatment resistance, and reduced survival [122,123,124,125,126].

### 5.2. Strategies to Target FAP

*Antibodies and Immunoconjugates*: Early attempts to target FAP involved the murine monoclonal antibody F19, which selectively recognized FAP on stromal fibroblasts in epithelial cancers [127]. Its humanized version, sibrotuzumab, entered clinical trials and showed a favorable safety profile in colorectal and lung cancers, but failed to demonstrate meaningful clinical efficacy [128]. To improve efficacy, newer approaches utilize antibody-drug conjugates and radioimmunoconjugates. Agents like αFAP-PE38 and radiolabeled antibodies (e.g., ^177^Lu-ESC11, ^177^Lu-ESC14) have shown the ability to deplete FAP^+^ stroma and suppress tumor growth in preclinical models [129,130]. Immunotoxin αFAP-PE38 was studied in the metastatic breast cancer mouse model [129]. ^177^Lu-labeled ESC11 and ESC14 have been tested in melanoma xenograft models [130]. In parallel, bispecific antibodies and fusion proteins are being developed to pair FAP recognition with immune modulation. Examples include RG7386 (FAP/DR5), RO7300490 (FAP/CD40), and FAP-4-1BBL, which co-engages T cells to enhance CD8^+^ T-cell activation and promote tumor regression [131,132,133,134]. FAP-4-1BBL was studied in preclinical rhesus monkey model with colorectal cancer and human patients with epithelial ovarian cancer or NSCLC [133,134]. These advanced constructs demonstrate how antibody formats can be engineered to overcome the limitations of early monotherapies, offering targeted cytotoxicity and immune activation by leveraging the tumor-restricted expression of FAP.

*Small Molecule FAP Inhibitors*: A major advance in targeting FAP has been the development of small molecules to inhibit FAP, including the quinoline-based FAP inhibitors (FAPI) [135]. Although early FAPI molecules had limited tumor retention time, structural improvements led to newer compounds such as FAPI-04, FAPI-46, and FAPI-74, which have continually improved the FAP binding capability of these molecules. Among these, FAPI-46 offers longer tumor retention and better imaging contrast, while FAPI-74 is suitable for broader clinical use due to dual labeling with ^68^Ga and ^18^F [136,137]. These agents have shown superior lesion detection across various cancers (e.g., sarcomas, head and neck, pancreatic) compared to FDG-PET [138]. However, their short biological half-life limits therapeutic applications, although newer versions have improved tumor retention time compared to the original compounds. Current efforts are exploring FAPI-drug conjugates and radioligand therapies to extend their role beyond diagnostics [139]. Li et al. have reviewed and summarized the clinical trials evaluating FAPI-04, FAPI-46 and FAPI-74. Ongoing clinical trials of FAPI-46 include breast cancer (ER-positive, lobular, and triple-negative cancer) [140], lung cancer [141], pancreatic/bile duct cancers [142], hepatocellular carcinoma, cancer of unknown primary [143], prostate cancer, sarcoma [144], and ovarian cancers [142].

*Peptides*: Peptide ligands offer an alternative to small molecules for FAP-targeting, with enhanced versatility and stability. Among these, FAP-2286, a cyclic peptide optimized for dual diagnostic and therapeutic use, has shown high binding affinity and metabolic stability in preclinical studies [145]. Its radiolabeled form, ^68^Ga-FAP-2286, demonstrated sustained tumor uptake in a Phase I trial, supporting its role in baseline imaging and therapy monitoring across several solid tumors (e.g., breast, colorectal, sarcoma) [146]. The therapeutic counterpart, ^177^Lu-FAP-2286, is under active investigation in the LuMIERE Phase I/II trial, with early data suggesting good tolerability [147,148]. Ongoing studies aim to refine dosing and assess clinical efficacy in advanced tumors. For example, ^68^Ga-FAP-2286 was employed as an imaging agent in various solid tumors [147], breast, bladder, prostate, colorectal, head and neck, pancreatic, sarcoma, cholangiocarcinoma, and lung cancers [149]. In terms of the safety profiles and clinical trials outcomes of different FAP-targeted radionuclide therapy, Privé et al. have thoroughly reviewed and compared FAPI-04, FAPI-46, FAP-2286, SA.FAP, ND-bisFAPI, PNT6555, TEFAPI-06/07, FAPI-C12/C16, and FSDD [150]. Additionally, in a single center, prospective study (64 patients with 15 types of cancer, 19 patients underwent paired ^68^Ga-FAP-2286 and ^68^Ga-FAPI-46 PET/CT) assessing the diagnostic accuracy of ^68^Ga-FAP-2286 to detect primary and metastatic lesions in patients with various cancer types by comparison with 18F-FDG and ^68^Ga-FAPI-46, researchers concluded that ^68^Ga-FAP-2286 and ^68^Ga-FAPI-46 yielded comparable clinical performance, especially in visceral and bone metastases, the quantitative tumor uptake of ^68^Ga-FAP-2286 was not inferior to that of ^68^Ga-FAPI-46 in the lung, liver, peritoneum, or bone. In one metastatic cholangiocarcinoma patient ^68^Ga-FAP-2286 PET/CT detected more subcutaneous metastases than ^68^Ga-FAPI-46 did [151]. Palihati et al. have recently reviewed ongoing clinical trials evaluating fibroblast activation protein targeting [152].

*Other FAP-Targeting Strategies*: Additional approaches have been developed to exploit FAP biology, including cellular therapies, vaccines, and prodrugs. FAP-targeted chimeric antigen receptor (CAR) T cells have shown promise in preclinical models by remodeling the tumor stroma and enhancing immune responses, with early clinical trials indicating acceptable safety [153,154,155,156,157]. These cells have been studied in human lung cancer xenografts and syngeneic murine pancreatic cancers [158,159]. Another phase I clinical trial studied F19-based CAR-T-cells in malignant pleural mesothelioma patients [155,156]. DNA vaccines against FAP reduced tumor growth and stromal collagen in animal models, improving drug delivery and reducing fibrosis without systemic inflammation [160,161]. They have also been studied in multidrug-resistant murine colon and breast carcinoma [160]. Enzyme-activated prodrugs like FAPα-activated vinblastine prodrug Z-GP-DAVLBH selectively disrupt tumor vasculature and have led to complete tumor regression in resistant models [162]. These innovative approaches highlight the expanding potential of FAP as a versatile therapeutic target.

An overview of representative agents, clinical trial status, and key findings across the major FAP-targeting strategies is summarized in Table 2.

### 5.3. Application of FAP-Targeting

Due to its selective expression in CAFs, minimal presence in normal tissue, and critical role in tumor progression, FAP has emerged as a promising clinical target, driving the development of novel strategies for imaging, radioligand therapy, and targeted drug delivery.

*Imaging*: Given its high expression in CAFs and minimal presence in healthy tissues, FAP has emerged as a promising target for tumor imaging. While CT scans have traditionally been used to visualize masses [167], PET imaging enables non-invasive in vivo visualization of dynamic inflammatory and cellular processes, enabling the visualization of stromal remodeling and therapeutic response across both oncologic and fibrotic contexts [168]. Compared to conventional ^18^F-FDG PET, long considered a cornerstone of oncologic imaging [167], FAP-targeted tracers offer simplified patient preparation, flexible imaging windows (10 min to 3 h post-injection), favorable biodistribution with low background uptake, and rapid renal clearance with minimal uptake in healthy tissue [112]. However, FAP imaging may also detect activated fibroblasts in non-oncologic conditions, including sites of fibrosis and inflammation [169].

Clinical studies have shown that FAPI PET often outperforms ^18^FDG PET in desmoplastic tumors [170]. In early-stage lung adenocarcinoma (stage IA), ^18^F-FAPI-04 PET/CT demonstrated higher SUVmax and tumor-to-background ratios than FDG, with tracer uptake correlating with FAP expression on immunohistochemistry [171]. Moreover, in patients with lung cancer evaluated using both tracers, ^68^Ga-FAPI PET/CT has been shown to detect a greater number of suspected nodal, pleural, bone, and intracranial metastases compared with ^18^F-FDG PET/CT [172]. In esophageal cancer, ^68^Ga-FAPI PET detected all primary cancers and additional nodal metastases missed by CT [173]. Systematic reviews in breast and gastric cancers confirmed that FAPI PET frequently identified more lesions, with superior sensitivity for nodal metastasis and recurrent disease [174,175,176]. Similarly, meta-analyses in hepatobiliary and pancreatic cancers showed FAPI PET outperforms ^18^F-FDG in nodal, liver, and distant metastasis detection [177]. In sarcomas, ^68^Ga-FAPI-46 PET demonstrated 96% sensitivity, with uptake correlating with FAP expression and altering staging or management in ~30% of patients [178]. Further studies have highlighted diagnostic advantage of FAPI PET in colorectal [179], ovarian [180], peritoneal [181], and bladder cancers [182], often with significant impacts on clinical decision-making.

The integration of FAP-targeted imaging into clinical practice will require the development of standardized guidelines for patient selection and scan interpretation. While ^18^F-FDG PET remains the current standard in oncologic imaging, FAP PET offers several advantages in specific clinical contexts. For example, tumors characterized by a dense desmoplastic reaction, such as sarcomas, pancreatic cancers, and lung adenocarcinomas, demonstrate robust FAP expression which results in high tumor-to-background contrast that may surpass the performance of FDG PET [170]. Additionally, FAP PET may outperform FDG in tumors with inherently low metabolic activity, such as gastric and pulmonary adenocarcinomas, where FDG uptake is limited and disease burden may be underestimated [171,183]. FAP imaging can also offer an advantage in anatomical regions with high physiological FDG uptake that may obscure malignancy, such as in hepatic tumors [179].

**Figure 2:**
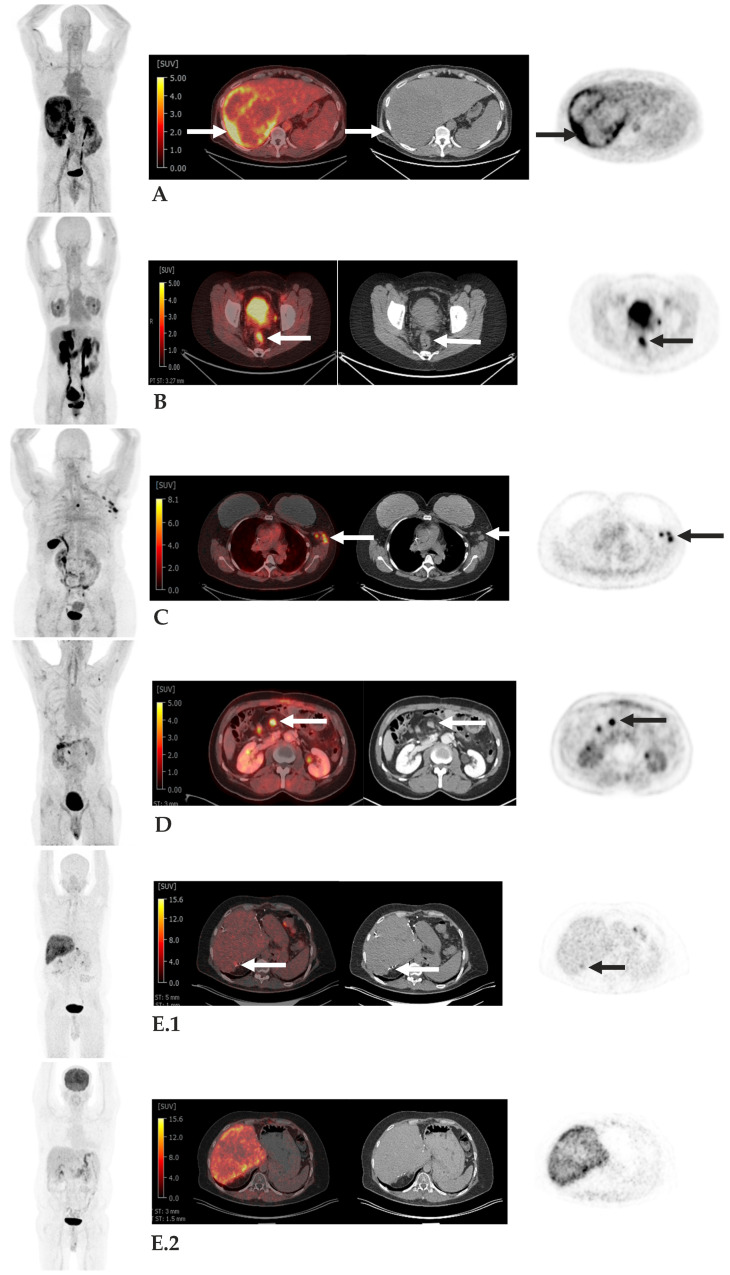
Examples of FAP PET Imaging. Case (**A**): 52-year-old man with hepatocellular carcinoma. Large right hepatic mass with intense peripheral FAPI uptake, Standard Value Uptake (SUV) up to 10.0 (arrows), consistent with patients known primary neoplasm. Case (**B**): 41-year-old man with FAPI-avid rectal cancer, SUV 6.7 (arrows), consistent with patients known primary neoplasm. Case (**C**): 52-year-old woman with breast cancer with FAPI-avid left axillary nodes, SUV up to 11.1 (arrows), consistent with metastases. Case (**D**): 55-year-old man with pancreatic adenocarcinoma post Whipple procedure with FAPI-avid central mesenteric node, SUV 9.1, consistent with metastasis. Case (**E**): 56-year-old man with colon cancer metastatic to liver post hepatic resections with focal FAPI uptake along segment 7 wedge resection (arrow) (Panel (**E.1**)) without correlate on corresponding FDG PET/CT (Panel (**E.2**)), subsequently biopsy-proven metastasis. Disclaimer ID: Memorial Sloan Kettering Cancer Center.

However, careful interpretation of FAP PET is essential due to the potential for off-target uptake in pathologic tissue though non-malignant conditions. Because FAP PET targets activated fibroblasts and fibrotic tissue, uptake may occur in areas of non-malignant fibrosis or inflammation. For instance, in patients with extensive pulmonary fibrosis, inflammatory uptake can confound evaluation for lung cancer. Similarly, FAP PET may demonstrate uptake in regions of physiological tissue remodeling or chronic inflammation, including degenerative joint changes associated with aging, rheumatoid arthritis, myocarditis, and scar tissue [184,185]. Awareness of these potential pitfalls is critical to avoid false-positive interpretations and ensure accurate clinical assessment. Representative examples of clinical FAP PET imaging across tumor types, illustrating the utility and diagnostic contrast of FAP-targeted tracers, are shown in Figure 2.

*Theranostics*: Beyond diagnostic imaging, FAP-targeting agents have been applied in theranostics, pairing PET-based detection with radionuclide therapy. In early studies, ^68^Ga-FAPI-04 PET/CT was used to localize metastatic breast cancer lesions, followed by therapeutic administration of ^90^Y-FAPI-04. High uptake was observed in metastatic sites, and the patients experienced pain relief, supporting this therapy [186].

Subsequent early-phase studies have supported the feasibility of radioligand therapy (RLT). In a Phase I trial, ^177^Lu-FAPI-46 was administered to 18 patients with advanced, treatment-refractory FAP positive cancers, achieving stable disease in two-thirds of cases with minimal radiation to healthy tissues [187]. A separate case series of nine patients with high FAP expressing advanced solid tumors treated with ^90^Y-FAPI-46 reported favorable tumor uptake and disease control in ~50% of patients, although grade 3–4 hematologic toxicity occurred in four cases [188]. In another first-in-human study in patients with advanced adenocarcinoma (pancreatic, breast, rectal, and ovarian), ^177^Lu-FAP-2286 showed prolonged tumor retention and high tumor absorbed doses, with low systemic exposure and manageable grade 3 adverse events [189].

FAP-targeting RLT has also been explored in radioiodine-refractory thyroid cancer. Treatment with ^177^Lu-DOTAGA.(SA.FAPi)_2_ in 15 patients who had progressed on standard therapies and demonstrated high FAP uptake on ^68^Ga imaging, reduced serum thyroglobulin and improved pain and performance scores, without significant hematologic, renal, or hepatic toxicity [190]. Encouraged by these findings, later-phase clinical trials are underway. The LuMIERE Phase I/II trial is currently evaluating ^177^Lu-FAP-2286 in advanced solid tumors, using paired ^68^Ga-FAP-2286 imaging for patient selection and dosimetry [147].

Together, these studies highlight the early but growing evidence that FAP-targeted theranostics may offer a safe, tolerable, and effective therapeutic option across multiple tumor types, particularly in settings where conventional treatments have failed.

*Drug Delivery*: Beyond theranostic applications, FAP-targeting has been explored in a range of drug delivery platforms, including prodrugs, antibody–drug conjugates (ADCs), and nanoparticle systems. These strategies aim to exploit the high expression of FAP in CAFs to achieve selective intratumoral drug activation or accumulation while minimizing systemic toxicity.

FAP-activated prodrugs remain inert in circulation but are enzymatically converted into active cytotoxic agents within the TME. In a 2014 study, the FAP-activated prodrug ERGETGP-S12ADT effectively suppressed tumor growth in mouse xenograft models of human breast and prostate cancers with efficacy comparable to docetaxel but markedly lower systemic toxicity [191]. Additional candidates have shown similar preclinical promise across tumor types, though none have yet reached clinical evaluation.

FAP-directed ADCs combine a monoclonal antibody specific to FAP with a potent cytotoxic payload. OMTX705, a humanized anti-FAP ADC, induced complete tumor growth inhibition and durable regression in patient-derived xenograft models, both as monotherapy and in combination with chemotherapy [192]. While early studies yielded positive results, translation into clinical trials remains pending.

Nanoparticle-based drug delivery platforms represent an emerging strategy for FAP-targeted therapy. In a 2020 study, lipid–albumin nanoparticles engineered to release paclitaxel in response to FAPα demonstrated enhanced tumor penetration and robust tumor regression in a pancreatic cancer mouse model, with minimal systemic toxicity [193].

**Figure 3:**
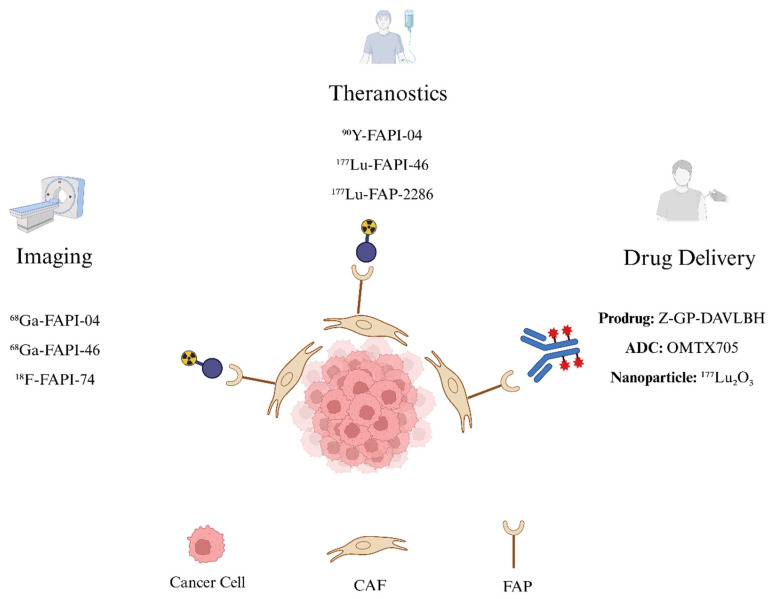
FAP-targeting Schematic. FAP expressed on CAFs is a promising oncologic target. FAP-targeting tracers labeled with positron-emitting isotopes such as ^68^Ga-FAPI-04, ^68^Ga-FAPI-46, and ^18^F-FAPI-74 enable diagnostic PET imaging, while FAP-targeting tracers labeled with β-emitting radionuclides such as ^90^Y-FAPI-04, ^177^Lu-FAPI-46, and ^177^Lu-FAP-2286 deliver targeted radioligand therapy. Beyond radiopharmaceuticals, FAP-targeting strategies also include drug delivery approaches such as prodrugs (Z-GP-DAVLBH), antibody–drug conjugates (OMTX705), and nanoparticles (e.g., ^177^Lu_2_O_3_). Figure created in BioRender.

Together, these approaches highlight the versatility of FAP as a tumor-specific target. While further clinical trials are necessary, these platforms offer a promising direction for expanding FAP-targeted interventions in oncology. A schematic overview of current FAP-targeting applications, spanning diagnostic imaging, radioligand therapy, and drug delivery platforms, is illustrated in Figure 3.

## 6. Targeting Other CAF Markers

While FAP remains the most studied CAF marker, additional fibroblast-associated molecules such as PDGFR, LRRC15, GPR77, and CD105 have emerged as promising therapeutic targets. These markers define distinct CAF subsets with specialized roles in tumor growth, immune evasion, and therapy resistance.

*Platelet-Derived Growth Factor Receptor (PDGFR)*: PDGFRα/β are tyrosine kinase receptors that regulate fibroblast proliferation via PI3K/Akt, MAPK, and ERK pathways [194]. Aberrant PDGFR signaling contributes to tumor progression, metastasis, and fibrosis, making it a useful therapeutic target for tyrosine kinase inhibitors and degradation agents [195]. A spatial transcriptomics study identified a novel PDGFRα^+^ITGA11^+^ CAF subset enriched in bladder cancer and associated with lymphovascular invasion and nodal metastasis [196]. Separately, a mechanistic study using the Cancer Genome Atlas (TCGA) data and breast cancer models demonstrated that PDGFRβ, along with caveolin-1 (Cav-1), promotes autophagy in CAFs through the mTOR/FIP200/ATG13 pathway and enhances tumor invasiveness via HIF-1α/MCT4/MCT1 signaling [197].

*Leucine-Rich Repeat-Containing Protein 15 (LRRC15)*: LRRC15 is upregulated in CAFs from breast, lung, pancreatic, and head and neck cancers. It fosters tumor progression, metastasis, and resistance to immunotherapy [198]. In ovarian cancer models, LRRC15 expression inhibited anoikis and promoted metastasis, which was suppressed by the LRRC15-targeted ADC ABBV-085 [199] Single-cell RNA sequencing of over 600 patient samples revealed that tumors with abundant LRRC15^+^ CAFs demonstrated poor response to anti-PD-L1 immunotherapy, highlighting their immunosuppressive role [46]. Mechanistic studies in pancreatic cancer and triple-negative breast cancer (TNBC) further implicated LRRC15 in fibroblast differentiation via TGFβR2 signaling and invasion through Wnt/β-catenin activation [67,200]

*C5a Receptor-Like 2 (GPR77)*: GPR77, often co-expressed with CD10, defines a CAF subset associated with cancer stem cell survival, chemoresistance, and tumorigenesis. GPR77 maintains NF-κB signaling through p65 phosphorylation and acetylation, sustaining an inflammatory CAF phenotype [69]. In gastric cancer patients undergoing neoadjuvant chemotherapy and gastrectomy, high post-treatment GPR77 expression correlated with worse tumor regression grade (TRG), indicating its potential as a predictive biomarker for poor therapeutic response [201].

*Endoglin (CD105)*: CD105 is a TGF-β co-receptor enriched in CAFs from breast, pancreatic, and cervical cancers. In pancreatic tumors, CD105^+^ CAFs secrete exosomes enriched in circAMPK1, which enhanced malignant progression [202]. In breast cancer, high CD105 expression in peri-epithelial CD105 expression, particularly in older women and BRCA1 carriers, correlated with increased risk of metastasis, notably to bone [199,203]. In cervical cancer, CD105^+^ stromal fibroblasts were linked to high VEGF-A expression, suggesting roles in tumor angiogenesis and vascular remodeling [204].

Table 3 summarizes key therapeutic strategies targeting non-FAP CAF markers, including PDGFR, LRRC15, GPR77, and CD105. For each marker, representative agents, mechanisms of action, relevant preclinical and clinical findings, and associated challenges are outlined.

## 7. Success Comparisons and Emerging Subtype-Specific Strategies

Theranostic approaches targeting CAFs, particularly FAP-positive subsets, have shown encouraging results but also revealed important limitations. For example, FAP-2286 has demonstrated strong tumor uptake and retention in both preclinical models and early peptide-targeted radionuclide therapy trials, yet variable patient responses highlight the functional heterogeneity of CAF populations [189,221]. Similarly, spatial single-cell studies in colorectal cancer revealed that enrichment of FAP^+^ fibroblasts and SPP1^+^ macrophages correlate with reduced benefit from anti-PD-L1 therapy, underscoring the immunosuppressive crosstalk between stromal and myeloid compartments [222]. These findings illustrate both the therapeutic promise and biological complexity of targeting FAP.

Clinical setbacks such as the failure of the monoclonal antibody sibrotuzumab in Phase II trials for metastatic colorectal cancer underscore the need to stratify patients based on CAF subtype. Incomplete enzymatic inhibition and intertumoral CAF heterogeneity likely contributed to its limited therapeutic benefits [223]. Emerging subtype-specific approaches also extend to non-FAP markers. For instance, LRRC15-targeting ADCs demonstrated enhanced efficacy in osteosarcoma cell lines with high LRRC15 expression. Notably, LRRC15-low cells could be sensitized to ADC therapy through TGFβ-induced re-expression, offering a strategy for overcoming resistance via tumor microenvironment modulation [93].

GPR77^+^ CAFs, driven by CCL18 signaling from TAMs, promote stemness and chemoresistance in breast cancer, but this effect can be reversed with anti-CCL18 therapy [215]. CD105, a TGF-β co-receptor, has been extensively studied in angiogenesis, particularly in renal cell carcinoma (RCC). TRC105, a monoclonal antibody targeting CD105, showed additive anti-angiogenic effects when combined with VEGF inhibitors in preclinical models. These promising results led to early-phase clinical trials evaluating this combinatory strategy [224]. Finally, beyond CAF-specific roles, PDGFR signaling has been implicated in both tumor stroma and bone remodeling, suggesting broader therapeutic opportunities [225].

## 8. Conclusions and Future Directions

Over the past decade CAFs have evolved from being viewed as passive structural elements to being recognized as dynamic modulators of tumor progression, immune suppression, and therapeutic resistance. This conceptual shift has profound implications for how we diagnose, monitor, and treat solid tumors. By integrating CAF biology into the broader framework of tumor biology, the stromal compartment is now seen not only as a therapeutic target but also as a diagnostic and prognostic resource.

The next decade of fibroblast-directed therapies is expected to rely on increased precision, integrating multi-marker and subtype-specific strategies rather than relying solely on single targets such as FAP. Preclinical and translational studies increasingly suggest that identifying distinct CAF subsets, including inflammatory (GPR77^+^), matrix-remodeling (LRRC15^+^), angiogenic (CD105^+^), and motile (FSP1^+^) fibroblasts, could enhance therapeutic efficacy while minimizing off-target effects. By distinguishing tumor-promoting CAFs from neutral or tumor-restraining populations, these approaches promise a more refined and clinically sustainable way of modulating the stroma.

This evolution is already reflected in the clinical pipeline. The LuMIERE trial, with an estimated conclusion date of 2028, is evaluating ^177^Lu-FAP-2286 in patients with advanced solid tumors, combining FAP-PET imaging for patient selection with radioligand therapy to measure safety, dosimetry, and early efficacy [147]. A novel FAP-activated prodrug of doxorubicin, named AVA6000, has demonstrated selective activation within the TME, which significantly reduces systemic toxicity while maintaining antitumor efficacy in FAP-rich models, and is currently in phase I clinical trials with an expected completion date in 2026 [226,227]. On the non-FAP front, the ABBV-085 tested an LRRC15-directed antibody–drug conjugate in sarcomas and other stromal-rich cancers, with endpoints including pharmacokinetics, safety, and preliminary response. The trial, which concluded in 2021, demonstrated ABBV-085 to be well tolerated at a biweekly dose of 3.6 mg/kg and showed early signs of efficacy, particularly in patients with osteosarcoma and undifferentiated pleomorphic sarcoma [211]. Meanwhile, newer FAP-targeted bispecific antibodies and imaging probes are entering early-phase trials to enable dynamic stromal profiling and patient stratification. Complementing these therapeutic developments, AI-enabled multimodal imaging and radiomics approaches are being explored to characterize stromal features, monitor therapy-induced remodeling, and predict immune related adverse events, with the goal of enabling real-time, response-adaptive treatment strategies [228].

The outlook for CAF targeting presents an exciting field. As this review shows, multiple clinical trials are in progress and may directly impact clinical practice. Growing knowledge of the subtypes of CAFs, as well as their intricate role in cancer biology, has unlocked the potential for more targeted approaches to therapies and diagnostics which have the potential to enter the clinic in the 2030s. Looking ahead, stromal targeting is expected to integrate into routine oncology practice for imaging and therapy. Advances in stromal imaging and spatial profiling are likely to support patient stratification and adaptive therapy monitoring in real time. As clinical trials indicate, CAF-directed interventions may increasingly be combined with immunotherapies, chemotherapies, and radiotherapies to break physical and immunologic barriers, thereby improving drug penetration, immune infiltration, and survival outcomes. Over the next decade, the stroma may shift from being seen as an obstacle to being recognized as a dynamic, targetable, and reversible element of solid tumor biology, central to precision oncology.

## Figures and Tables

**Table 1:** Summary of key cancer-associated fibroblast (CAF) markers, their associated subpopulations, functions, tumor-type specificity, and interpretative notes. (PDAC, pancreatic ductal adenocarcinoma; NSCLC, non-small cell lung cancer).

Marker	CAF Subpopulation	Function	Specificity/Note	Tumor-Type Specificity	Reference
FAP	Activated CAFs (broad)	ECM degradation, immune regulation, tumor progression	Low expression in normal tissues; target for imaging and therapy; not expressed in all CAF subsets	Broad across many tumors; PDAC, colorectal, breast, NSCLC, head and neck, skin, lung, endocrine/neuroendocrine cancers	[58,59,60,75]
α-SMA (ACTA2)	myCAFs	ECM remodeling and desmoplasia, contractility	Expressed in myofibroblasts; non-specific, also found in vascular smooth muscle cells, macrophages and pericytes	PDAC, breast, colorectal, lung, prostate, skin, renal, head and neck cancers	[56,57]
FSP1 (S100A4)	Broad CAF population	Cell migration, EMT, ECM remodeling and fibrosis	Also, in macrophages and endothelial cells; detects non-FAP+, Ki67− quiescent fibroblasts	Broad across many tumors; Breast, colorectal, gastric, pancreatic, prostate, ovarian cancer	[11,14,53,62,63]
PDGFR-α/β	iCAFs, general fibroblast pool	Proliferation, migration, cytokine signaling, angiogenesis	Also expressed by pericytes, adipocytes, and mural cells; subtype stratification context-dependent	PDAC, NSCLC, breast, lung, skin, prostate, colorectal, gastric, head and neck cancer	[14,53,65]
Vimentin	General fibroblasts	Cytoskeletal structure, EMT	Highly nonspecific; expressed in all mesenchymal and EMT+ epithelial cells, and leukocytes	All solid tumors (non-specific)	[54,66]
Podoplanin (PDPN)	Immune-modulatory CAFs	T cell exclusion, immune regulation	Present in lymphatic endothelial cells; upregulated in inflamed stroma	Lung, NSCLC, breast, colorectal cancers	[76,77]
LRRC15	TGF-β-responsive myCAFs	Matrix remodeling, immune supression	Subtype-specific; associated with poor prognosis; enriched in PDAC and NSCLC	Lung, breast, PDAC, NSCLC, sarcomas	[46,67,68]
GPR77 (C5aR2)	iCAF-like, chemoresistance-promoting CAFs	Cancer stem cell maintenance, inflammation	Associated with poor prognosis in lung and breast cancer; potential therapeutic target	Breast and lung cancers	[69]
MHC-II (e.g., HLA-DRA, CD74)	apCAFs	Antigen presentation, immunomodulation	Expressed without co-stimulatory molecules; limited T cell activation capacity	PDAC, breast cancer	[12,70]
CD-90 (Thy-1)	Various CAF subtypes	Adhesion, migration, fibrosis signaling	Expressed in multiple stromal and hematopoietic cells; interpretation context-dependent	Colorectal, pancreatic, breast cancers	[71,78]
Tenascin-C	Matrix CAFs	ECM reorganization, immune suppression	Expressed in wound healing and tumors; not exclusive to CAFs	Breast, prostate, PDAC, colorectal, glioblastoma	[72,73,76]

**Table 2:** Summary of major approaches FAP-targeting, including small-molecule inhibitors, peptide ligands, monoclonal antibodies and immunoconjugates, CAR-T cells, vaccines, and prodrugs. For each category, representative agents, clinical status, and key findings or limitations are reported.

Strategy	Representative Agents	Clinical Trial Status	Tumor Type	Key Findings/Limitations
Small molecules (FAPI ligands)	FAPI-02, FAPI-04, FAPI-46, FAPI-74	Multiple phase I/II studies [142,143,144]	breast cancer (ER-positive, lobular, and triple-negative cancer) [140], lung cancer [141], pancreatic/bile duct cancers [142], hepatocellular carcinoma, cancer of unknown primary [143], prostate cancer, sarcoma [144], ovarian cancers [163]	High tumor uptake and favorable T/B ratios; FAPI-46 shows longer retention; ^18^F-labeled variants enable multicenter use; short biological half-life remains a limitation [135,164].
Peptides	FAP-2286 (^68^Ga and ^177^Lu labeled)	Phase I [147]; Phase I/II LuMIERE trial	various solid tumors [147], breast, bladder, prostate, colorectal, head and neck, pancreatic, sarcoma, cholangiocarcinoma, lung cancers [149]	Demonstrated strong tumor uptake and favorable tolerability; under evaluation for therapeutic dosing and objective response rates; represents a true theranostic ligand [165].
Monoclonal antibodies/immunoconjugates	mAb F19, Sibrotuzumab, αFAP-PE38, ^177^Lu-ESC11/ESC14, bispecifics (RG7386, RO7300490, FAP-4-1BBL)	Early clinical trials [128,166] and multiple preclinical studies	αFAP-PE38: metastatic breast cancer mouse model [129], Sibrotuzumab: metastasized FAP-positive carcinoma patients, including colorectal carcinoma and NSCLC [128,166]. ^177^Lu-ESC11 and ESC14: melanoma xenograft models [130]. FAP-4-1BBL: preclinical rhesus monkey model with colorectal cancer, and human patients with epithelial ovarian cancer or NSCLC [133,134]	Safe but limited efficacy as monotherapy; conjugated formats (radioimmuno- and antibody-drug conjugates) and bispecifics show enhanced stromal depletion and immune activation [127,129,130,131,132,133,134].
CAR-T cells	FAP-CAR-T, F19-based CAR-T, Nectin4/FAP dual CAR-T	Phase I [156], preclinical and early clinical evaluation [157]	human lung cancer xenografts and syngeneic murine pancreatic cancers [158], F19-based CAR-T-cells: malignant pleural mesothelioma patients [155,156]	Reduced ECM deposition and tumor vasculature; suppressed tumor growth in murine models; first-in-human mesothelioma trial showed feasibility with local administration [154,155,158].
Vaccines	FAP-targeted DNA vaccines	Preclinical	multidrug-resistant murine colon, breast carcinoma [160]	Suppressed tumor growth and metastasis; reduced collagen deposition and enhanced chemotherapy uptake; recent study showed reduction in cardiac fibrosis without systemic toxicity [160,161].
Prodrugs	Z-GP-DAVLBH	Preclinical	various xenograft models [162]	FAP-activated prodrug disrupted tumor vasculature and induced complete regression in xenograft models; promising but not yet clinically tested [162].

**Table 3:** Summary of Therapeutic Strategies Targeting Non-FAP CAF Markers. This table outlines therapeutic agents directed at non-FAP CAF markers such as PDGFR, LRRC15, GPR77, and CD105. For each target, it highlights representative drugs, mechanisms of action, key preclinical or clinical findings, and current challenges. The comparison emphasizes the benefits and limitations of these approaches and underscores the need for marker-specific, selective therapies.

Target Marker	Examples of Drugs or Agents	Mechanism of Action	Preclinical and Clinical Outcomes	Challenges Present
PDGFR (α/β)	Imatinib [205], Sunitinib [206], Sorafenib [207]	Inhibits fibroblast proliferation [208], ECM remodeling, angiogenesis [209]	Mixed outcomes; some stromal suppression [210] but limited survival benefit [208]	Redundant pathways allow tumor escape [210]
LRRC15	ABBV-085 (antibody–drug conjugate) [211]	Direct cytotoxicity against LRRC15+ CAFs [212]	Preclinical: tumor regression in solid cancers [68], early clinical trials ongoing [213]	Subtype-restricted expression [214]
GPR77	Monoclonal antibodies (experimental) [201]	Targets CAFs supporting cancer stemness and chemoresistance [215]	Preclinical: reduced stemness, restored chemo sensitivity [69]	Still investigational [216]
CD105	TRC105 (anti-CD105 antibody) [217]	Inhibits CAF-mediated angiogenesis via TGF-β pathway [218]	Clinical trials: modest activity [219], limited benefit as monotherapy [220]	Best in combination with other therapies [220]

## Abbreviations

ADC, antibody–drug conjugate; α-SMA, alpha-smooth muscle actin; apCAF, antigen-presenting CAF; CAR-T, chimeric antigen receptor T cell; CAF, cancer-associated fibroblast; CD, cluster of differentiation; CXCL, C-X-C motif chemokine ligand; DOTA, 1,4,7,10-tetraazacyclododecane-1,4,7,10-tetraacetic acid; ECM, extracellular matrix; EGF, epidermal growth factor; FAP, fibroblast activation protein; FASL, Fas ligand; FDG, fluorodeoxyglucose; FSP1, fibroblast-specific protein 1; GPR77, G-protein coupled receptor 77; HGF, hepatocyte growth factor; HLA, human leukocyte antigen; iCAF, inflammatory CAF; IL, interleukin; JAK-STAT3, Janus kinase/signal transducer and activator of transcription 3; LRRC15, leucine-rich repeat-containing protein 15; mCAF, matrix-remodeling CAF; MHC-II, major histocompatibility complex class II; myCAF, myofibroblastic CAF; NSCLC, non-small cell lung cancer; PD-1/PD-L1/PD-L2, programmed death-1/ligands 1 and 2; PDAC, pancreatic ductal adenocarcinoma; PDGF, platelet-derived growth factor; PDGFR, platelet-derived growth factor receptor; PDPN, podoplanin; PET, positron emission tomography; RCC, renal cell carcinoma; RA, retinoic acid; RLT, radioligand therapy; TAMs, tumor-associated macrophages; TGF-β, transforming growth factor beta; TME, tumor microenvironment; TNC, tenascin-C; Tregs, regulatory T cells; VCAF, vascular CAF; VEGF, vascular endothelial growth factor; VIM, vimentin.
